# Tobacco policy in Israel: 1948–2014 and beyond

**DOI:** 10.1186/s13584-015-0007-x

**Published:** 2015-05-01

**Authors:** Laura J Rosen, Maya Peled-Raz

**Affiliations:** Department of Health Promotion, School of Public Health, Sackler Faculty of Medicine, Tel Aviv University, Ramat Aviv, Israel; International Center for Health, Law and Ethics and School of Public Health, University of Haifa, Haifa, Israel

**Keywords:** Tobacco control, Tobacco policy, Health policy, MPOWER, FCTC, Legislation, Israel, Smoking, Secondhand smoke, Tobacco smoke exposure

## Abstract

**Background:**

Tobacco is the only consumer product known to kill half of its users, and is a significant cause of death and disability to exposed nonsmokers. This presents a unique conundrum for modern democracies, which emphasize personal liberty, yet are obligated to protect citizens.

In Israel, the death toll in 2014 from smoking is expected to reach 8000 deaths; nearly a fifth of the population smokes, and over two-thirds of the population are exposed to tobacco smoke.

**Aim:**

This paper provides an overview of tobacco policy in Israel since the inception of the State, presents the development of the National Tobacco Control Plan, and recommends future actions.

**Methods:**

Sources for this article included the Knesset (Israeli Parliament) and Ministry of Health websites, Health Minister Reports to the Knesset on Smoking, and the scientific literature.

**Results:**

Israel has an impressive record on tobacco control policy, beginning with taxation in 1952, landmark smoke-free air and marketing legislation in the early 1980’s, tax increases and expansions of smoke-free air and marketing legislation in the ensuing years, and the addition of subsidized smoking cessation technologies in 2010. Until 2011, actions were taken by various organizations without formal coordination; since the passage of the National Tobacco Control Plan in 2011, the Ministry of Health has held responsibility for coordinating tobacco control, with an action plan.

The plan has been partially implemented. Smoke-free air laws were expanded, but enforcement is poor. Passage of critical marketing and advertising restrictions is stalled. Requested funds for tobacco control did not materialize.

**Recommendations:**

In order to prevent hundreds of thousands of preventable premature deaths in the coming decades, Israel should considerably strengthen tobacco control policies to include: guaranteed funding for tobacco control; strong curbs on advertising, promotion and sponsorship of tobacco and smoking products; public education; law enforcement; protection of children from exposure to tobacco; regulation of electronic cigarettes and other alternative harm-reducing products; tobacco control research; and systematic monitoring of, and periodic updates to, the National Tobacco Control Plan. Israel should also begin discussions of Endgame scenarios, and consider abolition of tobacco, as it continues its progress towards making smoking history.

## Background

Tobacco is the only consumer product known to kill half of its users, and is a significant cause of death and disability to nonsmokers exposed to tobacco smoke [1]. This presents a unique conundrum for modern democracies. On the one hand, tobacco consumption is initially a personal decision, the risks are well-known, and democracies are loath to regulate personal behavior even when it is known to damage the health of the individual. On the other hand, harm to society through premature death and disability of users and those exposed is enormous. Complicating the story are the enormous power of the transnational tobacco companies, the strength of the tobacco lobby in many countries, and the dependence of many governments on direct and indirect revenues from tobacco [2].

Israel, a modern democracy of just over eight million citizens [3], has witnessed a decline of more than 50% in the prevalence of cigarette smoking over the past 40 years [4]. Just under a fifth of Israeli adults (18.7%, 2013) currently smoke [4]; this is slightly higher than current levels in the U.S. (17.8%, 2013) [5], and Canada (17.3%, 2011) [6]. The change observed in Israel parallels that seen in other developed nations, as it places Israel squarely in the third to fourth phase of the epidemiologic curve of tobacco use, in which both male and female smoking levels are on the decline [7,8]. This decrease has occurred despite millions of New Israeli Shekels (NIS) of annual investment in tobacco advertising, sponsorship, and promotion by the tobacco industry, particularly by the transnational tobacco companies [4]. Though progress has been considerable, the harm of smoking in Israel continues: the death toll from tobacco use and exposure was estimated to be 10,000 Israelis in 2003, comprising 22% of mortality [9], and is expected to be about 8000 in 2014, more than the combined mortality from vehicle accidents, suicides, murders, obesity, lack of physical activity, and motor vehicle emissions combined [10]. This suggests that over the coming decades, several hundred thousand Israelis will die prematurely due to active smoking or exposure to tobacco smoke.

The aim of this paper is to explore Israel’s governmental tobacco policy, in the context of the continuing decline in smoking rates in the population. We explore two inter-related areas of action: one, the passage of piecemeal tobacco control legislation, and two, the complex, multi-faceted process of building, passing, and implementing an integrated National Tobacco Control Plan.

## Methods

We used the following sources of information: the Knesset (Israeli Parliament) website [11], which contains a complete record of all attempts (successful and unsuccessful) to pass tobacco regulation since 1999; Minister of Health (MoH) Reports to the Knesset, which since 2001 have provided an annual update on smoking prevalence and tobacco control actions; MoH website [12], which includes a record of all Ministry-issued directives since 1980; and articles published in the professional literature. We also conferred with individuals active in tobacco control in Israel, including professionals in the MoH.

We first present an overview of tobacco use and exposure in Israel during the years 1970–2013, and then identify players in tobacco control in Israel. We next focus on highlights in Israeli tobacco policy, utilizing the concepts of the World Health Organization’s (WHO) MPOWER structure [13]. MPOWER was a plan developed by the WHO to aid countries in reducing the demand for tobacco. We then describe the growth, development, and adoption of Israel’s National Tobacco Control Plan (NTCP). We conclude with recommendations for further action.

## Results

### Overview of tobacco use and exposure to tobacco smoke in the Israeli population

**Figure 1:**
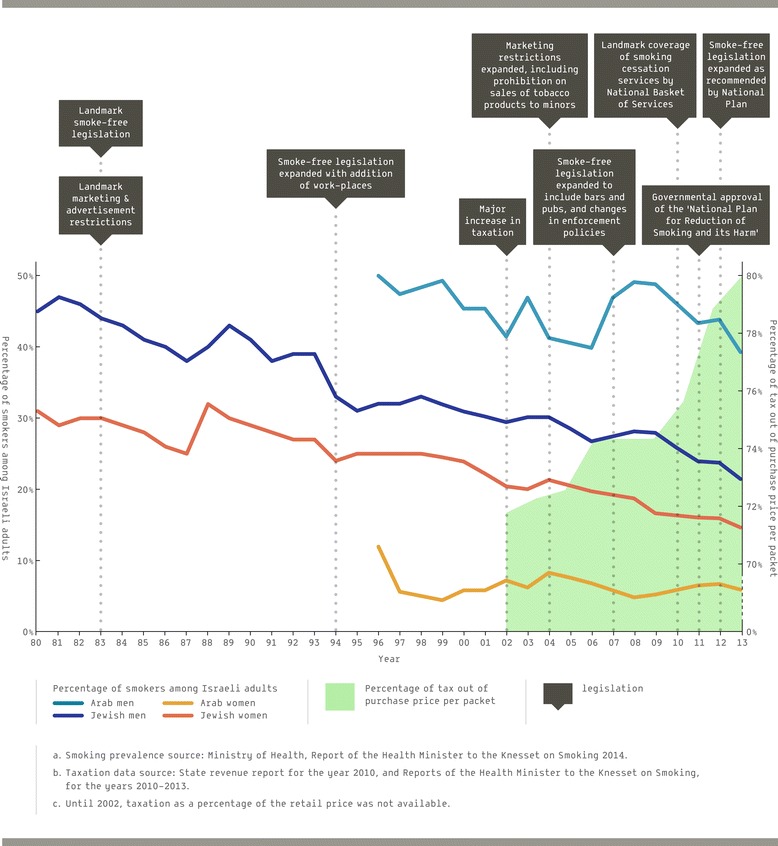
**Major tobacco policy landmarks, tobacco taxation, and population smoking prevalence and exposure 1948–2013.**

Published information on tobacco use in Israel among Jews dates back to 1972 [14]. At that time, 43% of Jewish men and 30% of Jewish women were smokers. Data on smoking among Arabs is available from 1996; at the time, smoking prevalence among Arab males was about 50% and among Arab females was about 12%. The most recent data, published in May, 2014, show that smoking among Israeli adults declined to 18.7% in 2013, and that declines were apparent in all four population sectors (Jewish men: 21.4%, Jewish women: 14.6%, Arab men: 39.2%, Arab women: 5.9%) [4] (See Figure 1).

Unlike information on active smoking, information on passive smoking has been collected only sporadically, and trends are unavailable. A study conducted in 2010 and based on a representative sample of Israeli adults found that roughly 70% of Israelis reported exposure at least weekly to tobacco smoke [15]. Results from the first Israeli Human Biomonitoring Study, conducted in 2011, showed that 62.2% of nonsmoking Israelis had urinary cotinine levels indicative of tobacco smoke exposure [16]. Data from 2013 showed that about 40% of nonsmoking Israeli adults reported exposure to secondhand smoke [4]. (P.11). In 2003–2004, nearly 86% of school children (7^th^-12^th^ graders) reported regular exposure to tobacco smoke, with 40% of them exposed on a regular basis at school [17]. Parentally-reported exposure of infants between 2009–2012 showed that 31.5% of infants aged 0–2 were exposed to tobacco smoke at least occasionally. (Jewish infants: 24.8%, Arab infants: 52.0%) [18] (p.176-178).

### Players in tobacco control in Israel

There are a number of players involved in tobacco control in Israel. Many of these are within the government: in addition to the MoH (including: the Department of Health Promotion and Education, which is located within the Public Health Services Branch, the Legal Department, the District Health Offices, and the Department of Maternal and Child Health), the following Ministries are involved: the Ministry of Finance, the Ministry of Education, the Ministry of Industry and Commerce (now, the Ministry of Economy), the Ministry of the Interior, as well as the municipal authorities, and the police. Public (non-profit) and voluntary organizations are also active. The Israel Cancer Association was the earliest leader in tobacco control, and has continued to spearhead activities for decades, running important media campaigns, initiating policy and legislative actions, promoting cessation, promoting smoke-free schools and teen smoking prevention, running lectures and seminars, and collaborating with other organizations [19]. The four Israeli HMOs, the Israel Association for Smoking Prevention, and the Healthy Cities Association have been active for many years. More recently, the Medical Association for Prevention of Smoking was established, as were several grass roots organizations, such as the Clean Air Organization and Haviv.

### Highlights of tobacco policy

In this section, we present, in chronological order, the highlights of tobacco policy and regulation from the inception of the State of Israel in 1948 through the present. We note that until the passage of Israel’s National Tobacco Control Plan in 2011, though the major tobacco control legislation was under the authority of the MoH, comprehensive tobacco-related policy was not officially under the jurisdiction of any single body or Ministry. Rather, it was promoted by various individuals and entities with varying agendas, albeit usually in collaboration with MoH personnel.

Tables 1, 2 and 3 present chronological details of policy efforts in the areas of 1- taxation, 2 - smoke-free public areas, and 3 – marketing, sales and promotion of tobacco products.

**Table 1:** **Changes to sales tax on cigarettes in Israel 1995-2013** ^**a**^

**Year**	**Direction**	**Tax basis**	**Percentile tax**	**Fixed tax (NIS per 1000 cigs)**	**But not less than (NIS per pack of 20 cigs)**	**Total % of tax out of purchase price**
**1952** ^**b**^	↑	Consumer price	53%			
**1995**	↑		53%	37.5		
**September 1996**	↑		53%	41.42		
**December 1996**	↑		55%	43.75		
**April 2002**	↑		58%	55.1		71.8% (based on the price of Marlboro cigs)
**December 2004**	↑		59%	62.5	5	72.3%
**June 2005**	↑		62%	49.49	6.2	72.6%
**May 2009**	↑		63%	62.5	8	74.4%
**June 2009**	-	wholesale price (consumer price + VAT)	225.7%	203.5	8	74.4%
**July 2009**	-		231.7%	194	8	74.4%
**July 2010**	↑		260.6%	214.5	9.5	75.7% (based on the calculated price of Time and Marlboro cigs)
**July 2012**	↑		278.6%	274.5	12.17	78.97%
**May 2013**	↑		270%	391.5	15	80.13%

^a^Base upon Israel’s State Revenue Administration’s annual report for the years 2011–2012.

^b^Between the years 1952–1995 tobacco taxing was mainly driven by budgetary needs, and fluctuated several times, until coming back to 53%.

↑Represents an increase in taxation.

**Table 2:** **Legislation regarding Restriction of smoking in public places 1983–2014**

**Main legislation**	**Supporting legislation**	**Year of revision**	**Details**
	Public health regulations (Prohibition of smoking in hospitals)	1982 (canceled 1983)	
**Restriction of smoking in public places act**		1983	Ban on smoking in cinemas and show theaters^a^, medical facilities^a^, communal areas in pharmacies, libraries^a^, educational facilities^a^, elevators, busses, taxis
	Restriction on smoking in public places regulations (Sign placement)	1984	“No smoking” sign required
		1988	Added banned venues - trains^a^, communal areas in supermarkets, diners^a^, coffee-shops^a^, restaurants^a^, gyms and kindergartens.
	Restriction on smoking in public places Regulations (Ushers)	1988	Property owner/holder allowed to appoint ushers to impose the “no-smoking” ban.
		1990	Added banned venues - communal areas in banks and post-offices
		1994	Added banned venues – workplaces^a^
		2001	Allowed “smoking area” changed into allowed “separate smoking room”, smoking entirely prohibited in hospital buildings, added banned venues - banquet halls^a^, malls^a^
	Public Health Regulations (Prohibition of Smoking in hospitals)	2004	Hospital directors must appoint ushers, charged with upholding the smoking ban
		2005	Appointment of “no smoking” ushers in hospitals.
**Prevention of smoking in public places and exposure to second-hand smoke Act (Name of act changed)**		2007	Added banned venue: bars^a^ and pubs^a^, duty of supervision and criminal responsibility imposed on property owner/occupant, municipal authorities required to appoint non-smoking supervisors, smoking prohibitions extended to apply to security forces.
	Criminal Procedure Order (fine offenses - preventing smoking in public places)	2007	Violation of the prohibition of smoking in public places is fineable without trail.
		2012	Added banned venues – entrances to medical facilities, partial outdoors of any food or beverage serving facility, enclosed bus stop, train station, outdoor swimming pool, public stair-cases, governmental offices in entirety, partial outdoors of banquet halls, places of religious worship including partial outdoors, youth center, nursing homes^a^.
		2014	Smoking in sports stadiums restricted to designated areas, of no more than a third of entire sitting area.

a = excluding designated smoking areas (until 2001)/ separate smoking room (since 2001).

**Table 3:** **Regulation of Advertising and Marketing of Tobacco in Israel 1983-2014**

**Main legislation**	**Supporting legislation**	**Year of revision**	**Details**	**Bill initiated by**
**Restriction on advertising and marketing of for-smoking tobacco products act**		1983	Banned advertisement via radio and television, in or on public transport, in public movie screenings, and in all printed material primarily intended for the consumption of minors; Restriction on Indirect Advertising, a limit placed on the number of ads allowed for each product per newspaper, and a ban on the use of human and animal figures as well as on the praising of smoking of itself in tobacco ads.	Ministry of Health
			Mandatory health warning added to all tobacco Product’s packaging.	
		1995	Mandatory health warning added to all tobacco adds	Parliament
		2001	Extension of the list of forbidden advertisement venues and means + interchangeable package health warnings allowed	Parliament
	Restriction on advertising and marketing of tobacco products regulation (wording of warnings)	2002	Interchangeable health warnings set	Ministry of Health
	Consumer protection order regulation (Marking of goods)	2004	Prohibition against the use of signs, marks or words the likes of “light”,” low tar”, “mild” and “ultra light”, on package, to indicate a “less harmful” product.	Ministry of Commerce
**Restriction on advertising and marketing of tobacco products act (Name of act changed)**		2004	Prohibitions extended to all tobacco products (not excluded to for-smoking products) + a prohibition set on the sale of tobacco products to minors	Ministry of Health
	Consumer protection order regulation (Advertisement and marketing venues directed at minors)	2006	A complete ban on tobacco product advertisement aimed at minors.	Ministry of Commerce
	Tobacco act	2006	Gave the MOH power to govern packaging of imported tobacco products	Ministry of Finance
		2008	A ban set on the selling of for-smoking products to minors	Parliament
		2011	Added ban on placing tobacco vending machines in the proximity of educational institutions	Parliament
		2014	Added ban on all placement of tobacco vending machines	Parliament

For each major item, we identify the relevant section of the WHO’s MPOWER framework [13], and summarize continued actions in each area which took place subsequent to the initial actions. In Figure 1, the major landmarks are juxtaposed graphically against population smoking prevalence and exposure.

### 1952-present: Taxation (MPOWER: raise taxes on tobacco)

Initiated in 1952, taxation of tobacco products is the oldest public health tool in the Israeli tobacco control scheme. It did not start as a public health tool, but rather as a fiscal one, and thus was placed under the sole authority of the Minister of Finance. The money raised by the tobacco retail tax was not designated for any particular use, and went into the general budgetary pool.

Due to the combination of pressure from the MoH to increase tobacco taxation for health reasons, and the pressure on the Treasury to balance a tight budget, the amount of all taxes imposed on cigarettes in Israel rose steadily, with a significant increase of over 10% between the years 2002–2013, to 80.13% today. This is just short of the EU average of 80.77% [4]. (p.81) Details can be found in Table 1.

### 1982 – present: Smoke-free laws (MPOWER: protect people from tobacco smoke)

Early legislation for restricting smoking in public places was initiated by the MoH in 1982 [20]. At the time, such actions were uncommon in other countries. The measure, applicable only to interior areas of hospitals, stipulated that smoking would be forbidden on hospital wards, allowed only in designated rooms which were not used for the treatment of patients, and that no-smoking signs should be centrally hung around wards, treating rooms and corridors.

This was followed by the landmark 1983 Restriction on Smoking in Public Places Act [21], which prohibited smoking in buses, taxis, pharmacies, libraries, educational and medical facilities (excluding designated smoking areas), elevators, movie theatres and other public halls. In a series of extensions to that law, smoking was prohibited in additional indoor places including public and private workplaces (excluding private rooms, not occupied by non-smokers), restaurants and snack bars, and most other indoor public places (although still allowing for the designation of separate, well-ventilated smoking rooms). Smoking was prohibited in elementary, middle, and high schools, (with the exception of designated teacher smoking rooms). A Ministry of Education directive issued in 2001 forbade smoking in school yards. Regulations pursuant to the law required “no smoking” signage.

In 2007, the Restriction on Smoking in Public Places Act underwent a major revision^a^. As manifested in the act’s revised name --“Prevention of Smoking in Public Places and Exposure to Second-Hand Smoke Act 1983” (henceforth Prevention of Smoking Act) – the legislation’s objective shifted from restriction to prevention. For that purpose, more rigorous enforcement tools were legislated. Responsibility for the implementation of the smoking ban was placed on the shoulders of property owners, as well as anyone else with jurisdiction over that place: the owner, a tenant or the authorized official in a corporation. Fines were increased for those who smoked in public places, and the owners became liable for relatively large fines (5,000 NIS). Ashtrays were banned in public places.

Smoking was also prohibited in bars and clubs, except for separate smoking rooms which were required to occupy less than 25% of the total area. All existing smoking restrictions were extended to army, police and prison services. Additionally, local authorities were required to appoint non-smoking inspectors.

In 2012, more venues were included in the smoking ban. The ban was extended to workplaces in their entirety (with the exception of a separate, well-ventilated, designated smoking room) and to all government buildings, with no exception for designated smoking rooms. Further, for the first time, some outdoor public areas were included in the ban: railway platforms, covered outdoor bus stops, outdoor swimming pools, 75% of the outdoor areas of bars, pubs, cafes, and party venues such as wedding halls. The area within 10 meters of entrances to hospitals and healthcare facilities were also designated as smoke-free. In June 2014, the law was further amended to allow for smoking only in designated limited areas in sports stadiums. A proposed amendment, restricting smoking in parks where children play [22], passed its first reading in the Knesset on January 8^th^ 2014. The bill was transferred to the Work, Welfare and Health committee of the Knesset, but has not been discussed since the first reading. Details can be found in Table 2.

### 1983 – present: Marketing regulations (MPOWER: warn about the dangers of tobacco)

The Restriction on Advertising of Tobacco Products for Smoking Act was enacted in 1983, with the primary aim of regulating the public’s exposure to tobacco advertising and secondary aim of providing a warning regarding the dangers of smoking.

Acknowledging tobacco companies’ superiority in advertising power, the Act, renamed the Restriction on Advertising and Marketing of Tobacco Products (RAMTP), as amended over the years, also endeavored to promote the dissemination of anti-smoking information by means of tobacco health warnings. Tobacco companies were required to place health warnings on the package of every tobacco product and on all tobacco advertisements. While in 1983, only one very small fixed warning text was set by the act^b^. Today, the law requires the use of 12 rotating warnings covering 32% of each major side of tobacco packages, in Hebrew on one side and in Arabic on the other, as well as the inclusion, in advertisements, of one of 13 rotating warnings, taking up 5% of the area of the ad [23]. ^c^Efforts through the years to obligate tobacco companies to divulge the contents of their products – by type and amount of each compound – have been unproductive. (See for example [24]) Similarly, attempts to expand the public’s exposure to health-promoting information, by requiring tobacco products’ packaging to carry details regarding hazardous components, have not been successful (See for example [25]).

### 1983-present: Marketing restrictions (MPOWER: enforce bans on tobacco advertising, promotion and sponsorship)

#### Advertising

The primary intent of the Restriction on Advertising of Tobacco Products Act (RAMPT) was to limit tobacco advertising. The original prohibitions were related to combustible tobacco products. The act was extended in 2004 to encompass all tobacco products.

As detailed in Table 3, the RAMTP initially forbade all tobacco advertising via radio and television, in or on public transport, in public movie screenings, and in all printed material primarily intended for minors.

Other restrictions included limits on the number of ads permitted for each product per newspaper, a ban on the use of models under the age of 40 in tobacco ads, which was later extended to the use of all human and animal figures, and a ban on praise of smoking in tobacco ads. The law was later extended to include a ban on indirect advertising of tobacco products, although sponsorship of cultural and social events was excluded from the term “indirect advertisement”, and so allowed, as long as the event was not child- or youth-designated.

In 2004, the consumer protection order was amended to prohibit the suggestion of harm-reduced products. The order specifically banned the use of the terms low- tar, ultra-light, light, and mild, as well as branding, graphic description or marking, suggesting the product was less harmful than others. In 2006 the consumer protection regulations were amended to completely ban any advertisement aimed at minors, regardless of mode of advertising.

Penalty for any breach of the act’s provisions was imposed on both the company that initiated the campaign and the advertising body.

#### Marketing

The marketing of tobacco products to minors has been forbidden since 2004 through an amendment to the RAMTP^d^. Vendors may require customers to present proof of age, and signage is obligatory. Penalties include high fines and business license revocation.

In 2001, the RAMTP was amended to authorize the MoH to forbid placement of tobacco vending machines in specified areas. This was further amended in 2011 to ban all vending machines. In the interim period, until entry of the ban into effect, regulations prohibited the placement of tobacco-vending machines within 1 kilometer of educational institutions. The ban on vending machines was challenged and later affirmed by the Israeli High court of Justice [26]. It went into effect in January 2014. Details can be found in Table 3.

### 2000 - present: Reports of the health minister to the Knesset on tobacco (MPOWER: monitor tobacco use and prevention policies)

In the year 2000, the Israeli parliament passed the Mandatory Reporting on Health Damages Caused by Tobacco Smoke Act. The Act required the annual reporting on prevalence of smoking, MoH tobacco control actions (legislation, media and educational campaigns), unsuccessful or incomplete legislative attempts (with explanations for failures); details regarding enforcement of smoke-free areas; and up-to-date scientific information regarding smoking health-hazards.

In 2007 the Prevention of Smoking Act was amended to require local authorities to report enforcement efforts of the ban on smoking in public places. These reports were then incorporated into the MoH’s annual report.

The first report came out for the 2001–2002 year. Reports have been published annually since that time.

### 2010 – present: Governmental subsidization of smoking cessation technologies (MPOWER: offer help to quit tobacco use)

Every year, from 2007 until the present, attempts have been made to add smoking cessation technologies to the National Basket of Health Services. The basket provides subsidized medications to all Israeli residents through the HMOs. The first success was achieved at the end of 2009. At that point, smoking cessation workshops became available without charge. Two smoking-cessation medications, one under patent (Champix), and one not under patent (Zyban) were included in the basket, with heavy subsidies, provisional on attendance at smoking-cessation workshops.

Effective Jan. 1, 2015, the widely-used and unpatented Nicotine Replacement Therapy (NRT) was added to the basket as a second-line tobacco cessation medication. Smokers attending cessation workshops, and for whom Champix or Zyban are contraindicated, are entitled to discount. Attempts to allow individuals to obtain Champix or Zyban with individual or phone counseling instead of group counseling, and to extend the subsidized period for tobacco control medications, have been unsuccessful.

### A chronological look at tobacco use, exposure, and policy

Figure 1 shows policy initiatives in the context of tobacco use over time. Annual data on smoking prevalence among adult Jewish men and women are presented from 1980. Data on smoking prevalence for Arab men and women were not regularly collected prior to 1996, and are shown from that year on.

Changes in tobacco taxation are shown from 2002, comparing the percentage of taxation on the price of a pack of 20 cigarettes^e^. Other major policy changes, including smoke-free regulations, marketing regulations, and the addition of cessation services to the National Basket of Health Services are also presented in chronological order.

As seen from the graph, Israel has experienced a major decrease in the prevalence of smoking in all population subgroups over the last decades, while simultaneously going through an increase in taxation as well as in the general scope of regulatory intervention. A surge in tobacco control policy activities can especially be detected since 2000.

### Israel’s National Tobacco Control Plan (NTCP)

**Figure 2:**
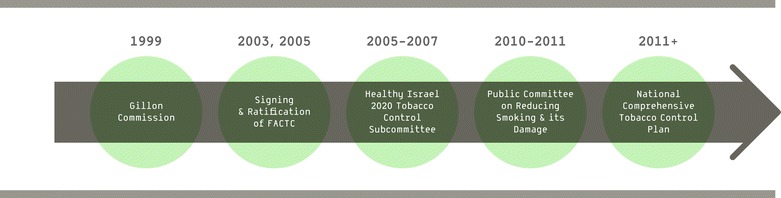
**Development of Israeli National Tobacco Control Plan.**

Israel’s National Tobacco Control Plan (NTCP), an integrated plan, was built in several distinct phases. The phases are pictured in Figure 2 and discussed below.

#### Phase 1: the Gillon Commission

The first attempt to coordinate actions regarding smoking occurred in 1998, as the inadvertent consequence of a different process. At the time, though the harms of active smoking were already well known and basic tobacco control legislation had been enacted, the MoH had not yet begun to systematically address tobacco use or exposure [27]. The Israel Physician’s Association petitioned Israel’s Supreme Court regarding the MoH’s perceived inactivity regarding smoking, and to force it/the government to regulate nicotine as a controlled substance under the Dangerous Drugs Ordinance. The MoH’s response was that the Dangerous Drugs Ordinance was not appropriate for regulating nicotine, and that a coordinated strategy to deal with tobacco use was needed instead. The Commission, headed by retired Judge Gillon, was appointed by the MoH and charged with making recommendations to decrease smoking and the harm caused thereby [28].

Topics discussed included the health and economic damage from smoking, the addictiveness of nicotine, and the dangers of exposing the public to secondhand smoke. The committee held extensive public hearings. The deadline for submitting a final report was extended seven times, but the report was not submitted.

#### Phase 2: Signing and ratifying the WHO’s Framework Convention for Tobacco Control (FCTC)

In 2003, the Ministry of Health and the Foreign Ministry, jointly initiated the signing of the WHO’s Framework Convention for Tobacco Control (FCTC). The FCTC, the world’s first health treaty negotiated under the WHO, was created as a response to the global tobacco epidemic, and sought to reduce both demand for, and supply of, tobacco products [29]. This treaty was ratified by Israel in 2005, formally committing the government of Israel to implement the FCTC provisions. These provisions included an overriding governmental commitment to decrease tobacco use and exposure, through regulation of demand for and supply of tobacco products.

#### Phase 3: Healthy Israel 2020

As with Phase 1, writing a strategy for a National Tobacco Control Plan in Phase 3 came as a byproduct of a different agenda.

Israel is a member of the European region of the WHO, and, as such, has various obligations, including the creation of health targets. In 1989, Israel produced its first set of health targets for the year 2000, in line with the WHO Health for All policy [30]. In 2004, MoH officials decided to update the targets. The initiative, dubbed “Healthy Israel 2020,” [“HI2020”] was built with inspiration from WHO Europe’s Health For All [31] and the US Healthy People 2010 [32]. In addition to creating a set of health targets, the endeavor expanded to identify evidence-based methods for the achievement of the targets and prioritization of strategies [33]. Tobacco was dealt with in a Subcommittee of the Health Behaviors Committee. The Tobacco Control Subcommittee defined its primary goals as the reduction of tobacco use and exposure in the population. Eight key elements were recommended as the basis of a National Tobacco Control Plan: creation of a central committee to coordinate efforts, taxation, legislation, enforcement, monitoring and research, community intervention, communication, and helping smokers quit [34]. However, HI2020 was not mandated to implement any particular strategies or programs.

#### Phase 4: National plan for reduction of smoking and its harm

In 2009, following the formation of a new government in Israel, both the political and professional leadership of the MoH changed. One of the first things which the MoH's new Director General did was to appoint a 5-member Public Committee to write a National Tobacco Control Plan (NTCP), which was entitled the National Plan for Reduction of Smoking and Its Harm. The idea was to create an operational plan based on the work done in the context of HI2020. The emphasis from the outset was on legislation, so as not to be dependent on the willingness of the government to fund activities.

Within six months, the preparation of the plan was complete. It included many elements from the 2020 plan, in particular, the establishment of a central body for tobacco control (now defined by the Public Committee as being within the MoH), creation of a comprehensive tobacco control policy with measurable goals, taxation, helping smokers to quit, protecting non-smokers from tobacco smoke exposure, use of media, and preventing youth initiation. Additional areas, which were adopted by the Public Committee but not covered by the 2020 plan, included the appointment of a committee to deal with environmental pollution from cigarette butts, and the development of a targeted plan for reducing the high smoking prevalence among Arab males.

As broad support of the national plan was considered essential for passage of laws which would likely be opposed by an active and vocal tobacco lobby, the plan was first presented as a whole to the Israeli Cabinet (“the government”) for approval; thereafter, the proposed legislation was submitted, as required, to the government committee on legislation, after having already been approved in principle by the government.

#### Phase 5: Approval in Knesset of the National Tobacco Control Plan and subsequent regulatory actions

The plan was approved by the Cabinet in May, 2011 [27]. For the first time in Israel, tobacco control made front-page news, and passage of the plan was accompanied by a lively public discussion in the media.

To date, several bills have been submitted to the Knesset in the wake of the NTCP. The first, submitted in May, 2012, concerned extensions to the law on smoking in public places. Most of these recommendations became law in July 2012.

In the summer of 2012, the MoH submited a bill which (amongst other proposed measures) would deem all advertising of cigarette products illegal, with limited exceptions to be determined. Larger warnings would be required on approved advertisments, and the MoH would be permitted to require graphic warnings on cigarette packages [35]. The bill was approved by the government and passed its first reading in a session of the 18^th^ Knesset. After the 2013 elections, the bill proceeded to the new 19^th^ Knesset’s Economics Committee for further deliberation and refinment, where it encountered major opposition. This opposition, led by tobacco lobbyists, and aided by the personal and political shift in the committee’s composition, led to extensive significant revisions in the original bill’s wording. In March 2014, in light of those changes, which the MoH viewed as contradicting the original logic and aim of the bill, the bill was withdrawn by the MoH. To date, no further progress has been made.

There has also been some action on the issue of electronic cigarettes. The MoH convened a committee to investigate the issue, which, in 2012, called for a five year moratorium, banning production, import and sales of e-cigs. The goal was to allow the scientific evidence on the harms and benefits of e-cigs to be accumulate before allowing entry of the product into the Israeli marketplace. The committee’s recommendation, as well as the MoH’s position, defining e-cigs as “medical products” – to be regulated through the Pharmacists Regulations [36], were presented to e-cigarettes manufacturers in May 2013, resulting in their prompt appeal to the Israeli High Court of Justice (HCoJ), requesting an injunction order against the MoH intention to ban production, import and sales of e-cigs through the Pharmacists Regulations^f^.

In December 2014 the Israeli High Court of Justice (HCoJ) rulled that the MoH is forbidden to restrict the production, import and marketing of e-cigs for recreational use through existing regulations, and may only attempt to do so by means of proposing a new law, for approval in the Knesset [37]. By contrast, marketing of e-cigs for medicinal use (e.g., smoking cessation or other medicinal purposes) requires authorization by the Pharmaceutical Division of the MoH, and is currently illegal, as such authorization has not been granted.

Anticipating this result, the MoH drafted and distributed such a bill for comments in September 2014, prior to the HCoJ’s ruling, proposing to ban the production, import, marketing and advertising of e-cigs and its related products, as well as applying the ban on smoking in public places to e-cigs [38]. The proposed bill has not yet been submitted to the Knesset.

## Discussion

Although impressive in its scope, the Israeli tobacco regulatory scheme until 2011 was not driven by a unifying or comprehensive strategy. Different governmental ministries regulated various aspects of use, manufacturing and import of tobacco. Restrictions on smoking in public places, as well as advertising and marketing restrictions, were under the regulatory authority of the MoH; tobacco taxation was under the jurisdiction of the Finance Ministry, and possible subsidization of tobacco manufacturing was under the jurisdiction of the Ministry of Industry and Commerce (now, the Ministry of Economy). There was often collaboration on main issues between Ministries, with advice being sought from the Ministry of Health. Yet, at times, the lack of a single authority led to inconsistent governmental actions and messages^g^.

This lack of central control – though not the authority of the various Ministries - changed with the creation and passage of the National Tobacco Control Plan in 2011. The passage of that plan brought two major changes. First, there is now a central coordinating tobacco control body in the MoH. That body, with three allotted full-time positions, coordinates tobacco control efforts throughout the government, informally coordinates some of the efforts in the public (non-profit), voluntary, and academic sectors, and is awaiting final approval to enforce tobacco control laws. Second, the coordinating body has a clear plan which it is systematically attempting to promote. As planned, taxation is being incrementally increased, for cigarettes and other types of tobacco products, and regulations extending smoke-free public places have received legislative approval. The submitted bill to limit advertising stalled and was unfortunately rescinded. The MoH has also initiated approaches not included in the NTCP, such as recommendations on policy regarding electronic cigarettes. Sporadic tobacco policy initiatives from outside the Ministry continue, most recently for fostering smoke-free public parks where children play^h^.

Although tobacco control activities have achieved significant improvement in smoking prevalence over the years, achievements elsewhere show that the potential exists for even greater progress. For example, smoking prevalence in 2013 in New York, Massachusetts, and Florida was about a sixth of the population, and in California was 12.5% [39]. All four of these states had exemplary tobacco intervention programs, and all have smoking rates which are lower than the national average [40,41].

Though Israel was once a leader in tobacco control, many countries currently have stronger tobacco control policies than does Israel. For example, though bans on advertising combined with point of sale restrictions have been shown to substantially decrease youth uptake, Israel lags behind on marketing, sponsorship, and promotion restrictions. By contrast, bans on local newspaper and magazine advertising exist in 45 of the 53 countries which form a part of the World Health Organization’s European Region; Israel is one of the few without such bans. Most (34/53) of these countries also prohibit internet advertising, though Israel does not. Sponsorship is also prohibited in a majority of these countries (29/53), though Israel allows it [42].

Further, some countries have created the goal of being tobacco free by a certain year; these include New Zealand (2025), Finland (2040), and Scotland (2034) [43].

Researchers have quantified effects of policy interventions on tobacco-related mortality and smoking-attributable deaths [44,45]. Jha emphasized the importance of taxation [45], while Levy, in an analysis of 41 countries, found that the largest number of smoking-attributable deaths was averted by taxation (47%), followed by smoke-free air laws (34%), warnings about harm (9%), cessation treatments (5%), and bans on marketing (4%) [44].

We propose recommendations to improve tobacco control policy in Israel, beyond the policies which have already been implemented (such as creation of a coordinating body for tobacco control and subsidization of tobacco cessation technologies) or which are being dealt with (such as increases in taxation).

### Recommendations

The recommendations are described below and summarized in Table 4. For each recommendation, we note if it was included in the National Tobacco Control Plan (NTCP), Healthy Israel 2020 (HI2020), the FCTC (with Article), or MPOWER (with appropriate letter underlined).

**Table 4:** **Recommendations for additional tobacco control policy measures in Israel**

**Recommendation**	**Supported by**
Guarantee funding for tobacco control	NTCP, FCTC Article 5
Place strong curbs on tobacco industry advertising, marketing, and promotion	NTCP, HI2020, FCTC Article 13, MPOWER
Educate the Israeli public about the dangers of smoking and exposure to tobacco smoke	NCTP, HI2020, MPOWER
Enforce tobacco control laws	NTCP, HI 2020, FCTC Articles 7 and 8, MPOWER
Protect children from tobacco smoke exposure	HI2020
Develop and implement a wise policy for e-cigarettes, other harm-reducing products, and alternative forms of tobacco	
Monitor the implementation and effectiveness of the NTCP and other tobacco control policies, and perform core tobacco control research	HI2020, FCTC (Article 5), MPOWER
Periodically update the NTCP, and include an Endgame strategy	FCTC (Article 5)

**NTCP:** Israel National Tobacco Control Plan.

**HI2020:** Healthy Israel 2020.

**FCTC:** World Health Organization’s Framework Convention on Tobacco Control.

**MPOWER:** World Health Organization’s suggested measures to assist in country-level implementation of effective interventions to reduce demand for tobacco.

### Guarantee funding for tobacco control (NTCP, FCTC article 5)

Though the NTCP was written with the hope of obtaining substantial funding, in practice this did not occur. Consequently, the actions to date have been concentrated on requirements and prohibitions, not accompanied by sufficient educational or enforcement measures (which require greater funding). This lack could be rectified by legislating that a small percentage of the billions of NIS (6.1 billion NIS in 2013) procured annually through tobacco taxation be funneled to tobacco control. To date, the Treasury has refused to consider this, as part of its economy-wide policy of opposing earmarked taxes. Another possibility would be to institute a “user fee” on the tobacco industry, such as was done by the U.S. Congress to fund the F.D.A Center for Tobacco Products.

### Place strong curbs on tobacco industry advertising, marketing, and promotion (NTCP, HI2020, FCTC article 13, MPOWER)

The limited extent of the advertising restrictions, and the existence of tobacco promotion and sponsorship, represent a severe limitation of current Israeli tobacco control policy. Tobacco companies have increased their funding for marketing, advertising, and promotion substantially in recent years, from 37.6 million NIS in 2007 to 61.3 million NIS in 2012 [4], p72-.73). Internet advertising is restricted in the same manner as other advertising, with restrictions on advertisements aimed at youth, but the restrictions are not enforced. Newspaper advertising remains legal, and the ongoing promotion and sponsorship of tobacco products severely limit the effectiveness of tobacco control measures.

### Educate the Israeli public about the dangers of smoking and exposure to tobacco smoke (NCTP, HI2020, MPOWER)

Significant educational effort requires extensive investment on part of the government, in terms of both money and expertise. Educational initiatives as well as resources may also originate in the voluntary sector, including the cancer associations and HMOs. In Israel, the Israel Cancer Association has taken an active role in educational efforts.

Data from European countries show that educational campaigns, which clearly explained that smoke-free legislation is intended to protect people against harm from exposure to secondhand smoke, can influence smokers’ support for tobacco control laws [46]. The fact that Israel’s smoke-free legislation is not well adhered to [47-49] may be partly attributable to the lack of media campaigns and other educational efforts accompanying the legislation. This lack is especially critical in view of the fact that over 60% of non-smoking Israelis are regularly exposed to tobacco smoke [15]. Particularly among the poorly educated, who have higher smoking rates [50], and less awareness of the dangers of smoking [51], knowledge imparted by educational efforts and media campaigns may increase support and cooperation with tobacco control policies.

### Enforce tobacco control laws (NTCP, HI 2020, FCTC articles 7 and 8, MPOWER)

In the absence of education regarding harm related to second hand smoke, and incomplete public compliance to no-smoking requirements, the need for effective enforcement is clear. Yet this also has not been given sufficient attention. Local authorities were found to implement incoherent and limited enforcement activity, while the municipal inspectors were found to be uncomfortable assigning high cost penalties [48]. Supporting these findings is the low number of fines given [4], implying a low level of law enforcement in areas which are legislated to be smoke-free but documented to have high levels of smokiness [47]. Indeed, the smoking ban’s enforcement by Israeli authorities has been so sparse that it prompted the Israeli Supreme Court to allow for “civil enforcement” of the ban, through private damage claims^i^.

Full realization of the Israeli smoking ban requires additional training of the ban’s enforcers, in order to increase the knowledge of inspectors regarding the hazards of second hand smoke, and to convince them that they are saving lives when they impose a fine [48]. In addition, intervention at the level of the Municipalities and monitoring inspector behavior could be helpful.

In 2013, a Smoking Prevention Division was established inside the MoH’s department for enforcement and supervision. Amongst other activities, the division is planning to train and educate local smoke-free inspectors on the importance of the proper application of their authority [4]. [pp. 62–63] However, the legislation to allow MoH employees themselves to enforce smoke-free laws has not yet been finalized.

### Protect children from tobacco smoke exposure (HI2020)

Children are especially vulnerable to the hazardous effects of tobacco smoke exposure [1] and need special protection, whether in public or private spheres. Current laws against smoking in schools are not enforced, and teacher smoking rooms remain legal. There are no laws in Israel preventing smoking in cars carrying children, in spite of the high levels of tobacco smoke exposure in vehicles where smoking takes place [52,53]. Such laws have been recommended by major organizations, including the U.S. Environmental Protection Agency [54], the American Lung Organization, and the U.S. Institute of Medicine [55], and have been passed in places such as Ontario, Canada and Oregon, U.S. [56] Policies which protect children at home, in cars, in school, and in public places should be carefully considered, in light of the potential to protect future generations and the complexity of regulating the private sphere.

### Develop and implement a wise policy for e-cigarettes, other harm-reduction products, and alternative forms of tobacco

There are many alternative and emerging forms of tobacco and smoking around the world. Because the magnitude and scale of risk differs with different forms of use, some professionals argue that harm reduced products should be encouraged. Others maintain that abolition of tobacco is the most practical path to improved population health [57]. Snus, widely used in Sweden but banned from other European countries, is credited with very low levels of tobacco-caused disease in Sweden [58]. (p. 76, p.198) In recent years, electronic cigarettes (e-cigarettes) have dominated the harm reduction debate. The tobacco control and public health communities abroad and in Israel are divided as to whether e-cigarettes represent a less dangerous alternative to smoking, with the potential to save lives and improve health, or whether it will re-normalize and re-glamorize smoking, thus acting as a gateway to cigarette smoking, and ending decades of progress in tobacco control [59]. Though a complete picture of the short and long-term risks from e-cigarettes, including their potential effect on population-level smoking of combustible products, is unknown, the present data indicate that e-cigarettes are far less deadly than traditional combusted cigarettes [60].

In Israel, a wise policy for recreational use of electronic cigarettes would include: restrictions on advertising, marketing and promotion (including prohibition of e-cigarettes sales to minors and a complete ban on internet advertising); quality assurance; appropriate warnings (particularly so as to prevent deaths from ingestion of e-liquid, and clearly inform the public of known and unknown risks); and disclosure of ingredients. Policy should be reexamined as new information becomes available.

The current policy of an unrestricted electronic cigarette market (excluding for medicinal purposes) ensures that market forces, with capital gains as the primary endpoint, dominate the landscape. This will continue until the government takes strong policy action.

### Monitor the implementation and effectiveness of the NTCP and other tobacco control policies, and perform core tobacco control research (HI2020, FCTC (Article 5), MPOWER)

At present, there is no formal plan for scientifically evaluating the effectiveness of the NTCP or other tobacco control policies in Israel, nor are there dedicated funds for this or for core tobacco control research. A small amount of research has been conducted on assessment of the laws enacted, primarily by academic researchers [47-49]. The MoH does internally review progress on implementation of the plan, but this information is not available to the public. The annual report of the Health Minister is submitted to the Knesset and discussed there, but does not specifically address evaluation of the NTCP. Cigarette smoking behavior is currently monitored by the MoH with its Knowledge, Attitudes, and Behavior (KAP) survey. A stronger research plan would include monitoring of: all elements of the NTCP; use of tobacco in any form and smoking products, including electronic cigarettes; public opinion and social norms regarding tobacco and tobacco control policies; objective measurement of tobacco smoke exposure among Israelis; tobacco industry activities, and content of tobacco and smoking products. The Population Assessment of Tobacco and Health (PATH), which is being conducted in the US [61], is an important example of core tobacco control research. This type of research would carefully monitor changes in use of various tobacco and smoking products by the Israeli public, and identify areas for intervention.

### Periodically update the NTCP (FCTC (article 5)) and include an endgame strategy

The NTCP, while based on the important sources of tobacco control both within and without Israel, is a document based on information and challenges relevant at the time it was written. New issues – such as electronic cigarettes – have emerged since then; new information is available on the implementation and failure of some of the NTCP’s components; and innovative approaches to tobacco control, such as plain packaging in Australia, are being discussed and implemented elsewhere. “Endgame” scenarios, in which cigarette smoking and tobacco use is drastically reduced into a marginal activity by a small percentage of the population, largely through innovative regulatory actions, have received a fair amount of attention [62]. Some researchers have even called for abolition of smoking [57] to battle the enormous toll of tobacco on society. These issues need addressing now, and others will arise in the future. We recommend that a periodic review of novel approaches and issues be conducted, in light of tobacco control progress in Israel and elsewhere.

## Conclusions

Israel has an impressive record of tobacco control policy, which includes steadily increasing taxation, comprehensive smoke-free laws, heavily subsidized cessation services, partial advertising restrictions, and a recently-adopted National Tobacco Control Program. Though once fragmented and sporadic, tobacco policy today in Israel is coordinated within the MoH, and has a comprehensive plan which it is promoting. Intermittent initiatives unrelated to the NTCP continue. That said, the death toll in 2014 from smoking is expected to exceed combined mortality from vehicle accidents, suicides, murder, obesity, lack of physical activity, and motor vehicle emissions; nearly a fifth of the population smokes, with smoking among Arab males close to 40%; existing smoke-free laws are poorly enforced, most of the nonsmoking public suffers from regular tobacco smoke exposure, internet advertising is nearly unrestricted, e-cigarettes for non-medical use, both with and without nicotine, are unregulated, and the tobacco industry invests enormous sums in advertising, marketing, and promotion in Israel. In order to prevent hundreds of thousands of future premature, preventable deaths, is time to begin to discuss Endgame scenarios, and to put the possiblity of abolition on the table.

## Endnotes

^a^The amendment followed Israel’s ratification of the Framework Convention on Tobacco Control, and was likely a reaction to Israel’s Supreme Court’s judicial criticism, in Civil Appeal Permit 9616/05 **Shemesh v. Fokacheta** (5/7/06), on the lack of enforcement of the no-smoking ban, as designed up to that time.

^b^The warning stated that “The MOH states that smoking is harmful to people’s health”.

^c^These include such warnings as “Medical research shows that smoking causes impotency” and “When you smoke you harm those close to you”.

^d^Under the Israeli law a “minor” is a person under the age of 18.

^e^Based on the calculated pack price for Marlboro and Time cigarettes.

^f^The e-cigs companies’ main claim was that e-cigs do not fall under the term “medical product”, thus falling out of the MoH’s authority to ban production, import and sales of medical products, through the Pharmacists Regulations.

^g^A good example of this can be found in the Ministry of Industry and Commerce’s (MoCI) decision to subsidize the operation of a planned tobacco factory near the city of Safed. The decision was based on socio-economic considerations which are made at the discretion of the MoCI: the goal of encouraging the development of work places in the periphery of Israel is among these. Nevertheless, the subsidizing decision stood in direct contrast to the anti-smoking message promoted by the MOH. The subsidizing decision was challenged in the Israeli Supreme Court by the Israeli Cancer Association. Although sympathetic to the petition, and critical of its consequent mixed message, the court refused to intervene in the decision, claiming that MoCI was authorized to make it, and autonomous in doing so. High Court of Justice’s case 194/88 **The Israeli Cancer Association v. The Director of the Investment Center** (7/9/1988).

^h^The formal initiator of that bill was the Israel Physician’s Association; the individuals involved were members of the Healthy Israel Tobacco Control Sub-Committee.

^i^In 2006 the Supreme Court ruled in favor of a plaintif, Irit Shemesh, who claimed to be harmed, along with her husband, by second hand smoke, while in a restaurant called Focacheta that allowed for smoking on its premises. Although offences under the Prevention of Smoking in Public Places and Exposure to Second-Hand Smoke Act of 1983 are criminal violations, and the law does not explicitly recognize personal compensable harm due to second-hand-smoke, the Supreme Court recognized the ban’s lack of enforcement as a basis for a personal harm claim, and stated that:

“The heaviness and slowness of the law enforcement authorities’ actions justify opening the door for ‘civil enforcing’, so that a concerned citizen who wishes to maintain his health as well as the public’s health would also be able to contribute to the common good.“Civil Appeal Permit 9616/05 **Shemesh v. Fokacheta** (5/7/06) Art. 4] The effectiveness of the court’s ruling as a deterrence tool has been limited, though, since not many citizens, injured by second-hand-smoke, are willing to take it upon themselves to go to court and endure a lengthy legal process, for the sake of being awarded the sum of approximately 1,500 NIS (about 429$) – the sum awarded to Mr. Shemesh”.

Nonetheless, this problem is expected to be mitigated, as the result of the Supreme Court’s 2013 ruling against the Bella Shlomkin’s club. A class action was brought against the club, based on the Shemesh v. Focacheta ruling, for harm caused to its patrons, due to the club’s failure to implement the smoking ban during a month in 2008. While the district court ordered the club to pay the Israeli Cancer Association 90,000 NIS for failing to ensure no smoking on the premises (Class Action (central district) 4398-09-08 **Litvin v. Bella Shlomkins** (Issued 26.1.2009)) the Supreme Court, sitting as the Court of Civil Appeals, decided to increase the club’s compensation payment to 1.16 million NIS (about 331,700$). This sum calculates a compensation of 1,000 NIS per patron (estimating 1,160 patrons per month). (Civil Appeal 2150/11 **Litvin v. Bella Shlomkins** (Issued 6.6.2013).

## Abbreviations

MoH: Ministry of health
FCTC: Framework convention for tobacco control
MPOWER: WHO package to implement the FCTC
HMO: Health maintenance organization
WHO: World Health Organization
TSE: Tobacco smoke exposure
SHS: Secondhand smoke
